# The Effects of Interpregnancy Intervals and Previous Pregnancy Outcome on Fetal Loss in Rwanda (1996–2010)

**DOI:** 10.1155/2015/413917

**Published:** 2015-11-03

**Authors:** Ignace Habimana-Kabano, Annelet Broekhuis, Pieter Hooimeijer

**Affiliations:** ^1^Demography and Statistics, University of Rwanda, P.O. Box 117, Huye, Rwanda; ^2^Utrecht University, P.O. Box 80115, 3508 TC Utrecht, Netherlands

## Abstract

In 2005, a WHO consultation meeting on pregnancy intervals recommended a minimum interval of 6 months after a pregnancy disruption and an interval of two years after a live birth before attempting another pregnancy. Since then, studies have found contradictory evidence on the effect of shorter intervals after a pregnancy disruption. A binary regression analysis on 21532 last pregnancy outcomes from the 2000, 2005, and 2010 Rwanda Demographic and Health Surveys was done to assess the combined effects of the preceding pregnancy outcome and the interpregnancy intervals (IPIs) on fetal mortality in Rwanda. Risks of pregnancy loss are higher for primigravida and for mothers who lost the previous pregnancy and conceived again within 24 months. After a live birth, interpregnancy intervals less than two years do not increase the risk of a pregnancy loss. This study also confirms higher risks of fetal death when IPIs are beyond 5 years. An IPI of longer than 12 months after a fetal death is recommended in Rwanda. Particular attention needs to be directed to postpregnancy abortion care and family planning programs geared to spacing pregnancies should also include spacing after a fetal death.

## 1. Introduction

An expert consultation organized by the World Health Organization in 2005 made an inventory of available research on births spacing. The experts recommended an interpregnancy interval (IPI) of at least 6 months after a miscarriage before attempting a next pregnancy, in order to reduce morbidity and mortality risks for mother, fetus, and newborn. An IPI of at least 24 months was recommended after a live birth, corresponding to a birth interval of at least 33 months. The consultation team also concluded that future research is needed on the mechanisms underlying the relation between interval length and pregnancy outcomes. More studies using datasets from both rich and poor countries could contribute to more in-depth knowledge [1]. Contradicting results of research on the effect of short intervals on the risk of adverse pregnancy outcomes [1–6] conducted after 2005 confirmed the relevance of these statements. The few studies on the effect of intervals after a previous pregnancy disruption show a large variation by country. DaVanzo and colleagues [4] emphasized that studies on the effects of interpregnancy intervals should take into account the outcome of the previous pregnancy.

Our study contributes to the debate on pregnancy loss and interpregnancy intervals (IPIs) in line with these recommendations. We focus on the effect of the duration of the IPI on pregnancy losses by combining the effect of the interval duration and the type of previous pregnancy outcome (pregnancy loss, live births that survived infancy or died in the first year), and controlling for important confounders. In this study, the term pregnancy loss includes all pregnancy outcomes (spontaneous and induced abortion, fetal death, and stillbirth) opposite to a live birth.

Fetal loss has got limited attention [7] compared to other issues, neither in the field of reproductive health nor in development debates among policy makers, nor in debates among scholars in population studies. This lack of attention is regrettable, because for many women the loss of a pregnancy is an emotional experience which affects their subsequent reproductive health and behavior. Fetal loss and stillbirths constitute the majority of the world's perinatal deaths and, yet, the absence of easy accessible and reliable secondary data on pregnancy loss is mentioned as a reason for neglecting the topic of fetal deaths by scientists [7, 8].

Reducing adverse pregnancy outcomes contributes to the health of the mother. Contrary to the reduction of maternal morbidity and of infant mortality, reducing pregnancy loss is not a policy objective but should become so in the future.

The outcome of our analysis will be discussed in the framework of results of a few [1, 4, 7, 9–11] available studies that followed the same approach by including the IPI duration and the previous pregnancy outcome to estimate the risk of a pregnancy loss. The majority of these studies focus on the effect of IPI duration on adverse pregnancy outcomes after a previous spontaneous or induced abortion while only a few have a broader perspective and include also other prior pregnancy outcomes (see Table 4). The studies differ on essential points, such as various types of mothers in the sample, nulliparous or multiparous women; various types of adverse pregnancy outcomes, pregnancy loss, preterm births, and low birth weight; reference group; categories of IPI; data collection method; and geographical region, which could contribute to explain the variety in the findings.

Only two studies, one from the USA and the other from Latin America, did not find significant associations between IPI duration and a fetal or neonatal death after a spontaneous or induced abortion [7, 9]. The other studies do find an association between IPI duration and adverse maternal and pregnancy outcomes after a previous fetal loss, but the associations between IPI duration and recurrent pregnancy losses are weak or nonexistent for late fetal deaths (stillbirths) [9–11]. Some results from USA and Scotland are even the opposite of what is expected on the WHO recommendations after early fetal deaths (miscarriages) [5, 6]. They find that, after a previous early fetal loss, short IPI intervals are associated with a higher likelihood on a live birth compared to longer IPIs. For Bangladesh [4], no differences between these likelihoods were found after a fetal loss according to IPI length except after very long IPI (>74 months).

However, the studies [10, 11] that focus on the association between IPI duration and the risk of a fetal death (and other adverse pregnancy outcomes) show higher risks of an adverse outcome after a fetal loss and a short IPI relative to a previous live birth combined with a healthy IPI. The meta-analyses [10] included many countries from all over the world and consequently examples with good and poor health care systems. A study on Sweden [12], a country with an advanced medical health care system, however, stated that risks are only found for long intervals and that the impact of short intervals may have been overestimated in other studies. From all those studies, we learn that it is important to include the outcome of the previous pregnancy in the analysis and to focus on several previous pregnancy outcomes when analyzing the effect of short IPIs on fetal losses. Therefore, we will follow this approach in our analysis about pregnancy loss in Rwanda.

Since 2000, Rwanda is experiencing a steady economic growth and after 2005 a rapid demographic and health transition [13, 14] thanks to the extension of access to reproductive health facilities [15]. The health service infrastructure, which was badly damaged during the civil war of the ninety-nineties, has been rebuilt to a large extent. At local level, community health centers are established and more than 45,000 community health care workers, male and female, have been trained to provide basic medical care and drugs and to give information on health matters. A third of those health care workers were trained in midwifery. The number of qualified medical staffs increased yet is still insufficient according to international standards: one medical doctor and one professional midwife per 16,500 and 23,400 inhabitants, respectively [16].

The government improved access to community health care also by introducing a community based insurance system. Today, more than 90% of the population participates in these* Mutuelles de Santé* “Health Mutualities” which give access to community level health services and, with additional payment, to a package of extra health care at district hospitals. This percentage of more than 90% explains an impressive increase in access to health care, given the fact that this percentage was only 7% in 2003. The prenatal checks and the costs of a normal delivery assisted by a nurse or midwife are covered by the basic health insurance schemes but exclude the 200 RwF per visit to a health center.

Yet, the health-seeking behavior among pregnant women still needs improvement although, according to the 2010 Demographic and Health Survey (DHS), less than two percent of the women did not have any antenatal medical test before the delivery. However, only 35 percent of them went for four antenatal checks, as recommended by the WHO. Most pregnant women got their first medical examination in the second trimester of their pregnancy and some even later [16–19].

## 2. Material and Method

The Demographic and Health Survey (DHS) is an internationally recognized data collection method that provides current and reliable data based on a national representative sample. Data from three successive Rwanda Demographic and Health Surveys (RDHS 2000, RDHS 2005, and RDHS 2010) were merged in this study to analyze the last pregnancy outcome of women within the DHS calendar periods of five years preceding the moment of interview.

Pregnancies and pregnancy outcomes that occurred in the eight months before the month of the interview were not included in our analysis to be sure that all pregnancies in the analysis had the same probability of ending in a pregnancy termination or a live birth after nine months. To identify the moment of the start of the last pregnancy, we used the detailed recording in the “calendar” of the DHS, which gives the pregnancy status for each month over a period of 59 months before the month of the interview. The nature and timing of the previous event are then defined. The exact months of all births, deaths, and pregnancy terminations are recorded in the DHS. The duration of the IPI is measured by subtracting the date of the previous pregnancy outcome from the date of the start of the last pregnancy.

The month of the previous outcome is registered at any time before the start of the last pregnancy. In total, 21532 women had at least one pregnancy outcome in the three reduced calendar periods before 2000, 2005, and 2010; for 3631 women, it was their first pregnancy (primigravida); for the other 17901 women, we calculated the date and type of the previous pregnancy outcome. In case of a long interpregnancy interval, this previous pregnancy outcome could have occurred before the five-year calendar period.

The DHS datasets enable the calculation of the exact date (in terms of month and year) of the events in the reproductive history of women in the sample if one combines answers to various questions in the questionnaire. We constructed Century Month Codes (CMC), the number of months elapsed since January 1900, of the pregnancy outcomes (pregnancy loss, infant death, and live birth) reported by the mothers to calculate the IPIs. In case of a live birth as last pregnancy outcome, the pregnancy was supposed to start 9 months before the CMC of the birth. In case of a fetal loss as last pregnancy outcome, the mother did report the duration of the pregnancy in months. Data from retrospective studies, like the Demographic and Health Surveys, are biased by errors due to memory lapses as the respondents have to report the number and date of the events in the past [20]. This is in particular the case when one asks for matters as pregnancy losses and induced abortions. Early pregnancy losses may not be noted or easily forgotten and induced abortions may not be reported as these are illegal in many societies. By focusing on the two last pregnancies of which the last one (and in many cases the previous as well) occurred during the calendar period, we reduced the risk of memory errors.

For our analysis, we calibrated a binary logistic regression model using the statistical package STATA 12. The dependent variable is the outcome of the last pregnancy (fetal loss coded as 1, live birth coded as 0). We checked whether a distinction between early and late losses gave different results, but this turned out not to be the case. To construct a more powerful model, we decided to take all fetal deaths together.

We defined the two main independent variables: length of the interpregnancy interval and previous pregnancy outcome as follows. Interpregnancy intervals (IPI) were calculated as the time between the outcome of the previous pregnancy that ended either in a pregnancy loss or live birth and the last conception. Short intervals are defined as shorter than 4 months or 4 up to 12 months after a previous pregnancy loss and shorter than 1 year or between 1 and 2 years after a live birth. A healthy interval after a live birth is an interval of at least two years and less than 5 years.

We categorized the live births of the previous pregnancy in two groups: infants that survived the first year of their life or infants that did not survive. The reason behind this categorization relates to the maternal depletion hypothesis and the quick return of the ovulation combined with the replacement strategy. In regard to the maternal depletion, the idea is to test whether a surviving breastfed infant will increase the depletion of maternal resources and therefore affect the survival of the next pregnancy that is conceived after a short IPI. Secondly, the death of the previous infant might be related to an unhealthy physiological status of the mother and the wish to quickly replace the infant, thus shortening the IPI.

We did not exclude multiple gestations, but we considered them as one birth. Subsequently, we constructed variables to represent the interaction between those two main independent variables in which we used different classifications for the IPI duration after the previous pregnancy outcomes. We tested for several confounding factors but, in the final model, included only four control variables that turned out to be of significance. The first is the inevitable biodemographic control variable age of the mother at conception which is an indicator for her physiological condition at the start of and during her pregnancy. We specified mother's age as a categorical variable to allow for nonlinear effects as we will focus on broad age categories and compare young (below the age of 21) and especially older mothers (over the age of 35) with women of a more optimal reproductive age. Age refers to the reproductive condition that contributes to a healthy pregnancy and the birth of a healthy infant. In particular, we want to test if older mothers have higher pregnancy loss risks compared to younger ones. The second control variable is the pregnancy wish. Question V228 of the DHS women's questionnaire asks the responding woman whether, at the time she became pregnant for the last pregnancy, she wished to become pregnant, she wished to wait until later, or she did not want to have any more children at all. Response categories included the following: wanting pregnancy then, wanting pregnancy later, wanting no more children, or unknown/vague answer. The latter category is used as a proxy for intended pregnancy losses together with the third included variable: place of residence that distinguishes between urban and rural residence.

The available dataset does not make a distinction between induced and spontaneous pregnancy losses, which is an omission seen in the different relations between the two types of abortions at the beginning of the IPI, length of the IPI, and pregnancy outcome found in other researches. However, we expect that Rwandan women will not easily indicate that they had an induced abortion as it is illegal, except when the physical health of the mother is in great danger. The study of Basinga and colleagues [21] estimated the rate of induced abortion in Rwanda in 2009 between 18 and 31 per 1,000 women in the 15–44-year-old group. Their study also revealed that the induced abortion rate was remarkably higher in the capital city Kigali compared to the situation in the provinces. In most African rural settings, induced abortion remains a taboo and social control in communities that watch over virginity as a core value, the reason why in particular rural women support legalization of abortion less compared to urban women [22]. As far as Rwanda is concerned, May et al. [23] stated that in the early nineties induced abortion did not occur traditionally in this country. For that reason, we expected that in case this situation changed during the last two decades abortions will be chiefly an urban phenomenon. We tried to control for it by including place of residence (urban versus rural) in the analysis. The percentages of unknown/vague answers in our dataset were, respectively, 27.9 and 23.6 for urban and rural women.

Finally, we included the year of the interview to check for changes over time in reproductive health: notably, the extent of the possible reduction of fetal mortality.  Table 1 gives the descriptive statistics of the research population.

## 3. Results and Discussion

The results presented in Table 1 illustrate that many Rwandan women still have to deal with pregnancy losses, the death of an infant, and unwanted pregnancies: 36 out of 1000 last pregnancies in our sample population ended in a pregnancy loss. Among the women with at least two pregnancies, 15 percent mourned a fatal outcome of the previous pregnancy: five percent had a pregnancy loss and nearly ten percent got a child that died in infancy. The results indicate also that the percentages of pregnancies ending in a pregnancy loss are the highest after an IPI shorter than 24 months that started after a pregnancy loss. Higher percentages of pregnancy loss than the mean of 3.6 per cent were found after a live-born infant that died in its infancy and an IPI of more than two years and after a surviving live birth and a very long IPI (>60 months).

The descriptive statistics in Table 1 show a modest decline of the rate of fetal losses during the period under study. For the three consecutive research periods, the rate of pregnancy loss diminished from 41 out of 1000 pregnancies (1996–2000) to 36 (2001–2005) and finally to 33 in the most recent period 2006–2010. It is difficult to assess if the total number of reported pregnancy losses in the three DHSs used in this study, 36 per 1000 pregnancies, is in line with expectations or not. It is a result of measurements over a rather diffuse period of time. The frequency fits within an indication given in medical literature that states that the number of fetal losses in the month after conception is high but that after a gestation of 8 weeks the loss is about 3 percent [21]. This could mean that women in Rwanda did not mention losses that occurred in the first one or two months of a pregnancy, when they were not fully aware of being pregnant. The early pregnancy losses reported in the DHS are probably underestimated as in poor countries the number of stillbirths (after a gestation of 28 weeks) is higher compared to that of rich countries and the stillbirth rate in countries in the central part of Sub-Saharan Africa varies between 25 and 40 or more per 1000 births [24]. With the number of early miscarriages added, the final rate must be even higher.

Women who were pregnant for the first time reported the highest percentage of wanted pregnancies (nearly 60%, see Table 2). Of all last pregnancies by the other women, only 40 percent were wanted at that time, while more than a third were unwanted or the mother gave an unclear answer (or answer not known). The cross tabulation presented in Table 2 shows that after a pregnancy loss or the loss of an infant a large portion of the women want to replace this loss. The percentages of wanted pregnancies extend the average of 40 percent. Very low numbers of wanted pregnancies are found among women who became pregnant within two years after the birth of the previous child that survived the first year of its life. Those two groups of women had liked to become pregnant later in time (indicated by 40 and 31%, resp.).

A large portion of the women became pregnant again before the recommended time (by WHO) for recovery was over. From the women whose previous pregnancy ended with a fetal loss, 43 percent were expecting a child again within half a year. For women who had a live-born child that died afterwards in infancy, 71 percent were pregnant again within two years after the last delivery, which could point at a replacement effect or at a lack of protection against pregnancy. For women whose child survived the first year of its life, this percentage was much lower (47%). This group of women is probably temporarily subfecund due to a longer amenorrhea period caused by lactation.

The constant (Table 3) reflects the risk of a pregnancy loss for the reference category: rural women in 2000, in age category of 21–35 years at the time of the last conception, whose last pregnancy was wanted and started after a healthy IPI (25–59 months), and whose previous pregnancy resulted in a child that survived its infancy. The estimate of the risk of experiencing a pregnancy termination for these women is very low (2%). The other variables Exp(*β*) give the odds ratios for women in the categories that deviate from the reference category.

Linking the risk of a pregnancy termination with both the outcome of the previous pregnancy and the length of the IPI (interaction variables) shows significant deviations from the risk estimated for the reference group: all except one point at a higher risk. The highest odds are found for women who became pregnant shortly after a previous pregnancy loss. Women who conceived again within 3 months after the previous pregnancy loss are 3.68 times more likely to lose the next pregnancy than the reference group with a healthy interval. The odds ratio is 2.648 for those that waited 4–12 months and even women who waited 12–14 months were almost twice as likely to lose the next pregnancy. Women with an IPI of more than two years after a previous fetal loss had a lower risk compared to the reference group, but the association is not significant. The higher odds of pregnancy loss for all groups with a previous pregnancy loss suggest that some women are prone to repeated losses, regardless of IPI duration. Repeated or recurrent pregnancy loss is phenomenon that puzzles medical experts like gynecologist already for decades and that is probably associated with more than genetic factors of the woman alone [25, 26].

After a live birth, regardless of whether the newborn survived its infancy or not, the likelihood of a pregnancy loss after an IPI considered as unhealthy (<2 years) is not higher compared to the reference group with a recommended IPI duration. For the mother that became pregnant within, respectively, one to two years after the previous birth, the signs of the coefficients are negative but only significant for women whose infant stayed alive and conceived within one year. Any pregnancy after a live birth seems to prepare for a successful next pregnancy, regardless of the interpregnancy interval.

This mechanism vanishes after some years, as an IPI of more than 5 years results in a substantial higher likelihood of a pregnancy loss (1.6 times more likely). This result is found in other studies as well. The risk of a pregnancy loss for mothers who are pregnant for the first time is of the same magnitude (1.5 times more likely). The physiological regression hypothesis states that after a very long IPI the body of a women has lost the beneficial physiological adaptations in her reproductive system that occur after a pregnancy [10, 12]. Her condition then resembles that of a primigravida.

According to the literature, a higher age at conception relates to lower fecundity and consequently longer IPI. Higher age is associated as well with physiological problems of the mother. This is reflected in the higher likelihood of a pregnancy loss (2.3 times more likely) for women who were older than 35 years when they became pregnant. The positive coefficients found for urban women and for women who gave a vague answer or did not answer the question on whether they wanted the last pregnancy could point at the occurrence of induced pregnancy terminations. As induced abortions are prohibited and a taboo, women who had an illegal abortion will probably answer evasively when asked for their pregnancy timing. The higher risk of pregnancy losses among urban women in our sample fits in with research findings by Basinga and colleagues [21] who calculated that induced abortions occur more frequently in the capital city of Kigali compared to other regions of Rwanda.

The finding that women who explicitly declared that the last pregnancy was not wanted have a significant lower risk of losing the next pregnancy gives food for thought. Maybe these are highly fecund women who become pregnant easily and therefore more often unwanted, and who do not encounter pregnancy problems.

We remark that the likelihood of a pregnancy termination decreased significantly between 2000 and 2010. For 2005, the sign of the coefficient (*β*) is negative, but the decrease is not significant. In 2010, however, the decrease is significant. Further analyses, not shown here, showed that this decrease pertained to late pregnancy loss only. This may be seen as an indication that improved health-seeking behaviour among pregnant women in particular during the second half of their pregnancy contributed to less pregnancy losses.

## 4. Conclusion and Policy Recommendations

The first important result of our analyses is that one needs to take the previous pregnancy outcome into account when estimating the effects of IPIs on the risk of a pregnancy loss. The second main finding is that negative outcomes (in terms of a higher risk of recurrent pregnancy loss) were found for IPIs up to 24 months after a prior pregnancy loss, a period four times as long as the recommended healthy IPI of only 6 months. In contrast, an IPI shorter than 2 years after a live birth does not seem to increase significantly the risk of a pregnancy loss. We are aware that a pregnancy loss is not the only possible adverse pregnancy outcome. Shorter IPIs than two years after a live birth do not give higher risks of a pregnancy loss, but they will affect other pregnancy outcomes such as preterm birth, low birth weight, low Apgar scores, and a higher neonatal death.

We found clear indications for negative effects of the replacement mechanism after the loss of a pregnancy. The replacement wish after a fetal death leads to shorter IPIs and therefore to a higher risk of another pregnancy loss [12]. Finally, the results of our study confirm the physiological regression hypothesis: a higher risk of a fetal death when IPIs are longer than 5 years. Also older women have a higher likelihood of a pregnancy loss compared to younger ones.

Our results are partially in line with the ones from DaVanzo and colleagues in Bangladesh [1, 11] based also on a general sample of women with all types of prior pregnancy outcomes. To avoid a higher risk of a next miscarriage or stillbirth also in Bangladesh, women should wait longer than the recommended 6 months (up to 15 months) to become pregnant again after a former pregnancy loss. The researchers found a significant increased risk of a pregnancy loss after a live birth and an IPI < 6 months. For longer IPI durations up to 74 months after a live birth, no significant higher risks of a pregnancy loss were found. After a duration of 74 months, the risk was again significantly higher.

Based on the results of this study on Bangladesh and ours on Rwanda, one could conclude that, in societies without an advanced health care system, the WHO recommendations concerning spacing after a fetal loss still count. Workers in the health care system should advise women, even if they are eager to become pregnant again, to take actions to prevent a quick new pregnancy and wait even longer than a year to become pregnant again.

The improvements in the Rwandan health care system between 2000 and 2010 and in particular the increased access to this system contributed to a lower pregnancy loss frequency. Probably, the increased antenatal checks during the last pregnancy period had an impact, as the significant decrease in pregnancy losses between 2000 and 2010 resulted in particular in fewer late fetal losses (after a pregnancy duration of 20 weeks). With a policy that recommends to women an IPI of at least a year to two years after a fetal death and more early pregnancy visits to the community health facility, a decrease in an early fetal death could be achieved as well.

## Figures and Tables

**Table 1:** Descriptive statistics: last pregnancy outcomes in percentages and in total numbers (pooled data from DHS 2000, DHS 2005, and DHS 2010).

Variable names	Latest pregnancy outcome	
	(*N* = 21532)	
	Pregnancy loss (%)	Total number
First pregnancy (primigravida)	3.3	3631
*Previous pregnancy outcome and IPI*		
Pregnancy loss		
IPI ≤3 months	10.6	161
IPI ≥4 months and ≤12 months	7.9	381
IPI ≥13 months and ≤24 months	8.0	217
IPI ≥25 months	4.7	149
Live birth (child died in infancy)		
IPI ≤12 months	3.3	662
IPI ≥13 months and ≤24 months	2.6	582
IPI ≥25 months	5.5	513
Surviving live birth		
IPI ≤12 months	2.3	1594
IPI ≥13 months and ≤24 months	2.7	5580
IPI ≥60 months	7.6	1314
IPI ≥24 and ≤59 months	3.5	6740
Age of mother at the latest conception		
20 years and younger	2.7	2075
36 years and older	6.8	4780
21 to 35 years	2.7	14677
Pregnancy timing		
Mistimed (later)	1.7	4116
Unwanted (no more)	1.4	2786
Unknown/vague answer	8.7	5246
Wanted (then)	2.2	9368
Type of place of residence		
Urban	4.2	4125
Rural	3.5	17407
Year of interview		
2000	4.1	6383
2005	3.6	6816
2010	3.3	8333
Total	3.6	21532

Sources: RDHS 2000, RDHS 2005, and RDHS 2010.

**Table 2:** Wanted the last pregnancy (in %) according to previous pregnancy outcome and IPI duration.

Prev. outc.	IPI	Vague	Unwanted	Mistimed	Wanted		Tot. no.
Primigr.	—	19.6	8.3	13.4	58.8	100.0	3631

Fetal loss	<3	24.8	14.3	14.9	46.0	100.0	161
	4 ≤ 12	20.2	12.9	12.9	54.9	100.0	381
	13–24	37.3	14.2	5.3	43.1	100.0	225
	25+	28.9	14.1	4.0	53.0	100.0	149

Live born died	≤12	23.9	9.8	13.7	52.6	100.0	662
	13–24	27.0	10.5	9.6	52.9	100.0	582
	≥25	31.6	11.8	6.3	49.7	100.0	513

Surviving live born	<12	28.4	11.2	39.7	20.7	100.0	1594
	13–24	21.1	13.5	31.5	34.0	100.0	5580
	25–59	25.1	15.2	14.0	45.7	100.0	6740
	60+	38.8	16.5	2.2	42.5	100.0	1314

Total		24.4	12.9	19.0	43.5	99.9	21532

Sources: RDHS 2000, RDHS 2005, and RDHS 2010.

**Table 3:** Binary logistic coefficients on the risk of pregnancy loss in Rwanda (pooled data 2000, 2005, and 2010).

Log likelihood = −3038.95	LR *χ* ^2^ (19)			642.2
	Prob. > *χ* ^2^			0.000
	Pseudo *R*2			0.096

Variable names	*N* = 21532	*B*	*P* > *z*	Exp(*B*)

Previous pregnancy outcome and IPI				
Previous live birth and IPI ≥25 and ≤59 months (Ref.)	6,740			
Pregnancy termination				
IPI ≥3 months	161	**1.303**	*∗∗∗*	**3.680**
IPI ≥4 months and ≤12 months	381	**0.974**	*∗∗*	**2.648**
IPI ≥13 months and ≤24 months	225	**0.663**	*∗*	**1.940**
IPI ≥25 months	149	0.102		1.107
Previous infant death				
IPI ≤12 months	662	−0.014		0.986
IPI ≥13 months and ≤24 months	582	−0.401		0.670
IPI ≥25 months	513	0.257		1.292
Previous surviving live birth				
IPI ≤12 months	1,594	**−0.410**	*∗*	**0.664**
IPI ≥13 months and ≤24 months	5,580	−0.083		0.920
IPI ≥60 months	1,314	**0.494**	*∗∗*	**1.639**
Primigravida	3,631	**0.400**	*∗*	**1.492**
Age of mother at the latest conception				
21 to 35 years (ref.)	14,677			
20 years and younger	2,075	−0.199		0.819
36 years and older	4,780	**0.843**	*∗∗∗*	**2.323**
Pregnancy timing				
Wanted (ref.)	9,368			
Untimed (later)	4,116	−0.108		0.898
Unwanted (no more)	2,786	**−0.751**	*∗∗∗*	**0.472**
Unknown/vague answer	5,262	**1.308**	*∗∗∗*	**3.698**
Place of residence				
Rural (ref.)	17,407			
Urban	4,125	**0.217**	*∗*	**1.243**
Year of interview				
2000 (ref.)	6,383			
2005	6,816	−0.117		0.889
2010	8,333	**−0.259**	*∗∗*	**0.772**
Constant		**−4.004**	*∗∗∗*	**0.018**

Significance: ^*∗*^<0.05,   ^*∗∗*^<0.01, and  ^*∗∗∗*^0.001.

Sources: RDHS 2000, RDHS 2005, and RDHS 2010.

**(a):** 

Study period/country	Sample	Effect of IPI	Reference category for statistical analysis	Controlled for
Wong et al. 2015 [6] USA (EAGeR trial) period 2006–2012	724 pregnant women with 1-2 prior pregnancy losses	No association between adverse pregnancy outcomes including pregnancy loss and IPI (<3 months or > 3 months)	Unknown	Demographic and reproductive history characteristics

Makhlouf et al. 2014 [7] USA, period 2003–2008	Nulliparous women 7681 primigravida 1240 with 1-2 previous spontaneous pregnancy losses (SAB) 817 with a previous induced abortion (IAB)	On fetal/neonatal death and other adverse outcomes after SAB and IAB No statistically significant difference for various IPI (<6, 6–12, >12 months) on risk of fetal loss and neonatal death	Primigravida Women with one previous SAB and IPI < 6 months	Maternal age, race, education, smoking, marital status, BMI, and use of vitamins C and E

DaVanzo et al. 2012 [4] Bangladesh (Matlab), period 1977–2008	9214 women with a miscarriage (spontaneous abortion prior to gestation of 28 weeks)	The shorter the IPI following a miscarriage is, the more likely the next pregnancy results in a live birth No significant effects of IPI duration on risks of a stillbirth Relative risk of a subsequent miscarriage increases with IPI duration	Women with a previous miscarriage and IPI of 6–12 months	Maternal age, education, gravidity, and calendar year

Love et al. 2010 [5] Scottish hospital data, period 1981–2000	30,937 women with a miscarriage in first recorded pregnancy	On miscarriage, ectopic pregnancy, IAB, and stillbirth Women with IPI < 6 months had less likely a miscarriage, highest sign Risk for women with IPI > 24 months No significant effect of IPI duration on risk of stillbirth	Women with a miscarriage and IPI of 6–12 months	Maternal age, socioeconomic status, year of first conception, and smoking

Conde-Agudelo et al. 2005 [9] Latin America period 1985–2002	258,108 women with a previous abortion	On fetal death and other adverse outcomes No significant difference of effect of IPI on fetal death or on neonatal death	Women with an IPI of 18–23 months	Maternal age, parity, education, smoking, marital status, BMI, year of delivery, hypertension, nbr antenatal checks, hospital type, and geographical area

IPI = interpregnancy interval, SAB = spontaneous abortion, IAB = induced abortion, and BMI = Body Mass Index.

**(b):** 

Study	Sample	Effect of IPI	Reference category	Controlled for
Conde-Agudelo et al. 2006 [3] Rich and poor countries from all over the world	Meta-analysis	On various adverse perinatal outcomes Less clear is the association between pregnancy spacing and the risk of fetal (and neonatal) death Curves suggest that IPI < 6 months and IPI > 50 months are associated with increased risks	Differs per included study	Various factors Not standard for previous pregnancy outcome

DaVanzo et al. 2007 [11] Bangladesh (Matlab) period 1982–2002	66759 pregnancies including multiple births of 28540 women	On various pregnancy outcomes An IPI < 6 months after a live birth lead to a 7.5 fold increase for miscarriages, and 1.6 fold increase for stillbirths. An IPI >75 months showed a less increased risk on fetal losses. After a fetal loss a higher likelihood of a subsequent same fetal loss regardless of the IPI duration Highest OR on a miscarriage after IPI < 6 months after a life birth	Women with live birth after IPI of 27–50 months	Socioeconomic status, maternal age, education both spouses, religion, and calendar year

Stephansson et al. 2003 [12] Sweden, period 1983–1997	410,021 women with two deliveries	Stillbirths and early neonatal death Previous reproductive history and maternal characteristics substantially confounded the association between IPI and risks of stillbirth Risks are only found for long intervals Role of short intervals may have been overestimated in previous studies	IPI of 12–35 months	Outcome of first pregnancy (*stillbirth/early neonatal death, preterm delivery,* etc.) Maternal age, education, presence of partner, country of origin, diabetes, hypertension, period of delivery, and smoking
